# A new species of *Aulacaspis* Cockerell, 1893 from China with a key to Chinese species (Hemiptera, Coccoidea, Diaspididae)

**DOI:** 10.3897/zookeys.619.9399

**Published:** 2016-09-27

**Authors:** Jiufeng Wei, Xiaopeng Jing, Hufang Zhang

**Affiliations:** 1 College of Agriculture, Shanxi Agricultural University, Taigu, Shanxi, China

**Keywords:** Aulacaspis, China, Diaspididae, Hemiptera, new species

## Abstract

A new species of armored scale insect, *Aulacaspis zunyiensis* **sp. n.** is described and illustrated from collections on cycads in China. A key to the *Aulacaspis* species known from China is provided.

## Introduction

The scale insects or Coccoidea are small, sap-sucking insects with at least 30 families and approximately 8000 species (Andersen et al. 2010; Hodgson and Peronti 2012), sister to Aphidoidea in the suborder Sternorrhyncha. Together with Psylloidea and Aleyrodoidea, they comprise the hemipterous suborder Sternorrhyncha (Kondo et al. 2008).

Diaspididae is the largest family of scale insects with over 2650 described species in around 400 genera as currently known (García et al. 2016). Conventionally, new species of armored scales are diagnosed based on extreme modification of the adult females, with the complete loss of legs, reduction of the eyes and antennae, and modification in the terminal segments of abdomen (Andersen et al. 2010). Many armored scale insects are agricultural pests and invasive species (Miller et al. 2005). The higher classification within the family is inconsistent, but two of the major subfamilies are the Aspidiotinae and the Diaspidinae.

The genus *Aulacaspis* Cockerell, 1893 is a large group of Diaspididae that belongs to the subfamily Diaspidinae. The genus was originally established by Cockerell (1893) with *Aspidiotus rosae* Bouché, 1833 as the type species. Since the introduction of the generic name *Aulacaspis*, many additional species have been described (e.g., Chen 1983; Chou 1982; Tang 1986; Takagi 1961, 1967; 1970; 1988; 1998; 1999; 2009; 2010a; 2010b; 2012a; 2012b; 2013; 2014; 2015; Williams 1988; 2010; Rutherford 1915; Robinson 1917; Takahashi 1931). The genus currently comprises 120 species (García et al. 2016; Takagi. 2012b; 2013; 2015), which occur in almost all zoogeographical regions except Antarctica (Suh 2013) and most are found in the Oriental and Palaearctic regions (Suh 2013). The species of this genus are associated with diverse plants and mostly feed on woody angiosperms (Takagi 2015). Some species of *Aulacaspis*, such as *Aspidiotus rosae* (Bouché) and *Aulacaspis yasumatsui* Takagi, are considered to be serious pests of ornamental plants (Milek et al. 2008; Miller et al. 2005; Watson and Marler 2014). China is the largest distributional region according to records of *Aulacaspis*, with 55 species having been reported in this country.

Recently, a new species of *Aulacaspis* was discovered in China, and it is described and illustrated herein, bringing the number of species recorded in this genus to 121, of which 56 are recorded from China. A key to the Chinese species of *Aulacaspis* is provided.

## Materials and methods

Infested plant samples were collected in the field. Permanent slide mounts of adult females from the samples were made according to Henderson (2011). The illustrations of the adult female are drawn from slide-mounted specimens, with the figure displaying the dorsal body surface on the left side and the ventral body surface on the right side. Enlargements of significant features are located around the body. The morphological terminology and measurements in the descriptions follows those of Miller and Davidson (2005). The abbreviations in the text refer to different pygidial lobes: L1 stands for the median lobes, L2 for the second pair of lobes, L3 for the third pair of lobes, and L4 for the fourth pair of lobes. All measurements are given in micrometres (µm). Measurements were made using the measurement tools NIT-Elements D.

The type series of the new species is deposited in the Insect Collection of Shanxi Agricultural University, Taigu, Shanxi Province, China.

## Taxonomy

### Aulacaspis

Taxon classificationAnimaliaHemipteraDiaspididae

Cockerell

Aulacaspis Cockerell, 1893: 180.

#### Type species.

*Aspidiotus rosae* Bouché: by subsequent designation by Newstead, 1901: 168.

#### Generic diagnosis.

**Female scale.** White, circular, exuviae located on front end.

**Male scale.** White, long and narrow, exuviae located on front end.

**Adult female.** Body shape varied, mushroom-shaped, fusiform or cuniform; derm membranous except for the margin of pygidium; prosoma swollen or wider than metathorax and abdomen, slightly squared in most species. *Cephalothorax*. Antennae each with a seta. Anterior spiracles each usually with a cluster of trilocular pores, posterior spiracles each with or without associated trilocular pores. Dorsal ducts present or absent on prosoma, scattered. *Pygidium*. Usually with three pairs of lobes (rarely with two or four pairs). Median lobes (L1) well-developed, much larger than lobules of lateral lobes, zygotic basally, without marginal setae between lobes. In general, L1 are divided into two types depending on feeding site: bark-type, where individuals occur on bark and L1 protrudes at the end of the pygidium; and leaf-type, on leaves and L1 is sunken into the end of pygidium. Second lobes (L2) much smaller than L1, bilobed, divided into inner lobule and outer lobule, outer lobule usually smaller than inner. Third lobes (L3) smaller than L2, bilobed, outer lobule smaller than inner. Fourth lobes (L4) present in some species and usually represented by serrations along the body margin. *Gland spines*. Marginal gland spines developed, present on lateral of abdominal segment II and III; usually single on abdominal segments V-VIII, but in some species there are two or more. Marginal gland spines becoming shorter to conical on anterior segments; in some species they are called gland tubercles. *Ducts*. Dorsum with double-barred ducts. Marginal macroducts of pygidium usually larger than dorsal macroducts. Dorsal macroducts forming submedial and submarginal rows on abdominal and pygidium, sometimes occurring in two sizes. Ventral microducts scattered. *Anal opening* situated at the center of the pygidium, small. Perivulvar disc pores in five groups.

#### Remarks.

Members of this genus, like other members of the subfamily Diaspidinae, have a pygidium with macroducts of the two-barred type, the second pygidial lobe bilobulate, and fringed plates absent between the lobes, but *Aulacaspis* is distinguished from other genera, especially *Chionaspis* Signoret, 1868 by having a remarkably swollen prosoma. Moreover, *Aulacaspis* lacks lateral macroducts and gland spines on abdominal segment I and on the thorax, present in these locations on *Chionaspis*. Furthermore, *Pseudaulacaspis* MacGillivray, 1921 is similar in features of the body, but can be distinguished by the presence of a pair of setae between the L1, which are absent in *Aulacaspis*.

### Aulacaspis zunyiensis sp. n.

Taxon classificationAnimaliaHemipteraDiaspididae

http://zoobank.org/D255B8CB-9DCB-4902-BBD1-2B12486EF0CF

Figures 1–9

#### Material examined.

Holotype and 11 paratypes, adult female. China: Guizhou Province. Zunyi city, longitude 106.9122, latitude: 27.7087, on *Cycas revoluta* Thunb, 17.vii. 2015, leg. Weijiufeng and Niu Minmin.

#### Description.

**Female scale.** Adult female cover convex, circular white; exuvia on front end. **Male scale.** Not recorded.

**Adult female.** Slide-mounted adult female 1150–1301 µm long (holotype 1246 µm long); widest part of body 901–950 µm wide (holotype 922 µm wide). Body outline fusiform, derm membranous except for pygidium. Usually widest at mesothorax, lateral abdominal and thoracic lobes well-developed; prosomatic tubercles slightly produced. *Cephalothorax*. Antennae each with one seta. Anterior spiracles each with 14–16 trilocular pores in a cluster, posterior spiracles without trilocular pores. *Pygidial lobes*. With three pairs of lobes; L1 well-developed, zygotic basally, much larger than lateral lobes; protruding from pygidial margin, with one deep notch and small serrations on outer margin and one obvious notch on apex. Without setae between median lobes; L2 bilobate, inner lobule rounded, much larger than outer lobule, outer lobule very small, smaller than L3, a pair of obvious paraphyses arising from the mesal margin of the L2 lobes. L3 bilobate, slightly smaller than L2. *Gland spines*. One present between L1 and L2, one present between L2 and L3, two present on abdominal segment VI, 3–5 on abdominal segment III, 4–5 on abdominal segment IV, 5–6 on abdominal segment V, 1–2 on abdominal segment II, 0–1 on abdominal segment I. Gland spines on segment I and II shorter than those on other segments. *Ventral gland tubercles* present on submargins of metathorax and abdominal segments I and II. *Ducts*. Marginal macroducts, of two-barred type, 12.8–16.3 µm long (holotype 16.0 µm long), absent between L1, one present between L1 and L2, two present between L2 and L3, two present on the abdominal segment V. Dorsal macroducts on pygidium and abdominal segments shorter than marginal macroducts; 8.5–10.2 µm long (9.6 µm long), of two-barred type, arranged segmentally in submedian and submarginal rows; submarginal dorsal macroducts present on abdominal segment II to V: 10–11 on segment II, 8–9 on segment III, 5–6 on segment IV, 4–7 on segment V; submedian dorsal macroducts present on segment II to V: 4–6 on segment II, 5–6 on segment III, 4–5 on segment IV, 3–6 on segment V. Lateral macroducts few, 5–7 in total, present between abdominal II and III, of which, 2–3 on segment II, 3–4 on segment III, smaller than dorsal ducts present on abdominal and pygidium. Ventral microducts scattered on pygidium, few. *Anal opening* small, in holotype posterior margin of anal opening is situated 155 µm from base of L1. Perivulvar pores in five groups, 13–16 in the median group, 30–35 in each of the anteriolateral and 29–30 in each of the posteriolateral groups.

**Figure 1–9.**
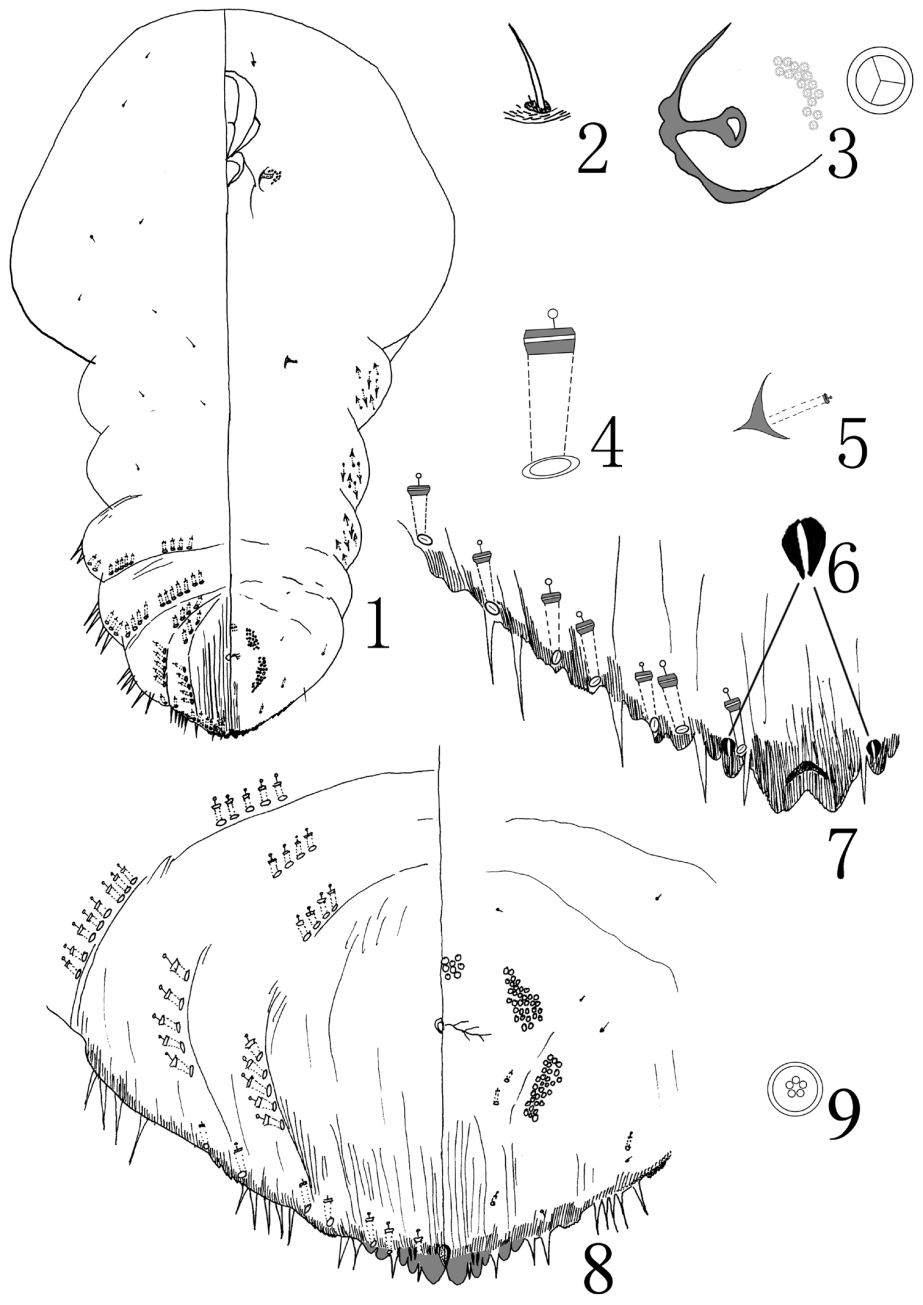
*Aulacaspis zunyiensis* Wei & Jing, sp. n., adult female; **1** habitus **2** antennae **3** anterior spiracle **4** detail of dorsal gland macroduct **5** gland tubercles **6** paraphyses **7** detail of end of pygidium **8** pygidium **9** quinquelocular pores.

#### Remarks.

This species is very similar to *Aulacaspis maesae* (Takagi, 1970) in body shape. But differs in having (character-states on *Aulacaspis maesae* in brackets): (i) posterior spiracle without trilocular pores (posterior spiracle with trilocular pores); (ii) dorsal macroducts absent from submedial region of abdominal segment VI (present); (iii) dorsal macroducts absent from submedial region of abdominal segment II (present).

#### Host plant.

*Cycas revoluta* Thunb.

#### Etymology.

The specific epithet is named after Zunyi, the type locality.

#### Distribution.

China (Guizhou).

### Key to adult female *Aulacaspis* Cockerell from China

(The descriptions of three species, *Aulacaspis aceris* Takahashi, *Aulacaspis formosana* Takahashi, and *Aulacaspis depressa* Zehntner are inadequate for inclusion in this key)

**Table:** 

1	Trilocular pores absent near each posterior spiracle	**2**
–	Trilocular pores present near each posterior spiracle	**9**
2	Dorsal microducts present on abdominal segment I , II, III	***Aulacaspis vitis* (Green)**
–	Dorsal microducts present on abdominal segment I, II, III	**3**
3	Dorsal macroducts present on submarginal and submedial area of abdominal segment II	**4**
–	Dorsal macroducts absent from submarginal and submedial area of abdominal segment II	**5**
4	Dorsal macroducts present on submedial area of abdominal segment VI	***Aulacaspis yunnanensis* (Feng)**
–	Dorsal macroducts absent from submedial area of abdominal segment VI	***Aulacaspis zunyiensis* sp. n.**
5	Dorsal macroducts absent from submarginal and submedial area of abdominal segment II	**6**
–	Dorsal macroducts present on submarginal and submedial area of abdominal segment II	***Aulacaspis pudica* (Ferris)**
6	With two or three dorsal macroducts present on submedial area of abdominal segment VI	***Aulacaspis fagraeae* (Green)**
–	With one or no dorsal macroducts present on submedial area of abdominal segment I	**7**
7	Dorsal macroducts absent from submedial area of abdominal segment VI	***Aulacaspis oblonga* (Chen)**
–	Dorsal macroducts present on submedial area of abdominal segment VI	**8**
8	With spur present on each of abdominal segment IV and V, submedian dorsal microducts present on abdominal II and III	***Aulacaspis calcarata* Takagi**
–	Without spur on abdominal segment IV and V, submedian dorsal microducts present on abdominal segment III, absent from abdominal II	***Aulacaspis schizosoma* (Takagi)**
9	Dorsal macroducts present on submarginal area of abdominal segment VI	**10**
–	Dorsal macroducts absent from submarginal area of abdominal segment VI	**11**
10	Submedial dorsal macroducts present on abdominal segment II, forming double row; dorsal submarginal macroducts present on abdominal segment II	***Aulacaspis difficilis* (Cockerell)**
–	Submedial dorsal macroducts present on abdominal segment II, forming single row; dorsal submarginal macroducts absent from abdominal segment II	***Aulacaspis altiplagae* Chen**
11	Submedial dorsal macroducts absent from abdominal segment II	***Aulacaspis litzeae* (Green)**
–	Submedial dorsal macroducts present on abdominal segment III	**12**
12	Dorsal macroducts absent from abdominal segment II	**13**
–	Dorsal macroducts present on abdominal segment II	**34**
13	Dorsal microducts present on submedial of abdominal segment I, II	**14**
–	Dorsal microducts absent from submedial of abdominal segment I and II	**15**
14	With four pairs of lobes on pygidium	***Aulacaspis madiunensis* (Zehntner)**
–	With three pairs of lobes on pygidium	***Aulacaspis ferrisi* Scott**
15	Both submedial and submarginal dorsal macroducts present on abdominal segment V and VI, forming double row	**16**
–	Both submedial and submarginal dorsal macroducts present on abdominal segment V and VI, forming single row	**17**
16	With four pairs of lobes on pygidium; L1 protrude the end of pygidium	***Aulacaspis wakayamaensis* (Kuwana)**
–	With three pairs of lobes on pygidium; L1 sunken into the apex of the pygidium	***Aulacaspis saigusai* Takagi**
17	Submedial dorsal macroducts forming double row on abdominal segment IV	**18**
–	Submedial dorsal macroducts forming single row on abdominal segment IV	**21**
18	Prosomatic tubercles robust; only 1 dorsal macroduct on abdominal segment VI	**19**
–	Prosomatic tubercles not discernible; with more than 2 dorsal macroducts on abdominal segment VI	**20**
19	Postsoma robust, with abdominal segment II strongly lobed out laterally; basal zygosis of L1 distinct	***Aulacaspis yabunikkei* (Kuwana)**
–	Postsoma slender, with the pygidium rather narrow; basal zygosis of L1 unconspicuous	***Aulacaspis alisiana* (Takagi)**
20	Anterior spiracles with about 20 trilocular pores; with 3 pairs of lobes on pygidium	***Aulacaspis sassafras* Chen, Wu & Su**
–	Anterior spiracles with about 70 trilocular pores; with 4 pairs of lobes on pygidium	***Aulacaspis tegalensis* (Zehntner)**
21	Submedial dorsal macroducts present on abdominal segment III, forming double row	**22**
–	Submedial dorsal macroducts present on abdominal segment III, forming single row	**24**
22	Dorsal macroducts absent from abdominal VI	***Aulacaspis robusta* Takahashi**
–	Dorsal macroducts present on abdominal VI	**23**
23	With more than three dorsal submedial macroducts on abdominal VI; anterior spiracles with 19 trilocular pores; the widest of body present on head	***Aulacaspis amamiana* Takagi**
–	With only one dorsal submedial macroducts on abdominal VI; anterior spiracles With 10 trilocular pores; the widest of body present on prothorax	***Aulacaspis ima* Scott**
24	Gland spines present on abdominal segment II	***Aulacaspis nitida* Scott**
–	Gland spines absent from abdominal segment II	**25**
25	Submedial dorsal macroducts absent from abdominal segment VI	**26**
–	Submedial dorsal macroducts present on abdominal segment VI	**28**
26	Prosoma well swollen; with more than 11 gland spines on abdominal segment III	***Aulacaspis sirodamo* Takagi**
–	Prosoma not swollen; with less than ten gland spines on abdominal segment III	**27**
27	Posterior spiracles with 4–5 trilocular pores; with slender paraphyses placed at base of L1	***Aulacaspis fuzhouensis* Tang**
–	Posterior spiracles with 2–3 trilocular pores; without slender paraphyses placed at base of L1	***Aulacaspis latissima* (Cockerell)**
28	Prosomatic tubercles robust	**29**
–	Prosomatic tubercles not discernible	**30**
29	With a pair of elongate scleroses on the base of L1; only 1 dorsal macroduct present on abdominal segment VI; anterior spiracles each with 4–5 trilocular pores	***Aulacaspis tubercularis* (Newstead)**
–	Without a pair of elongate scleroses on the base of L1 ; with 2–3 dorsal macroducts on abdominal segment VI; anterior spiracles each with 8–13 trilocular pores	***Aspidiotus rosae* (Bouché)**
30	Without dorsal microducts on prosoma	**31**
–	With dorsal microducts on prosoma	**33**
31	L1 almost parallel on inner basal margins, then strongly divergent to their apices; gland tubercles absent from segment I	**32**
–	L1 sunken into the apex of pygidium, forming a large notch at the apex of the pygidium; gland tubercles present on segment I	***Aulacaspis actinodaphnes* Takagi**
32	Only one submedial macroduct present on abdominal segment III; prosoma as broad as or slightly wider than postsoma	***Aulacaspis hedyotidis* (Green)**
–	With 2–8 submedial macroducts on abdominal segment III; prosoma swollen, distinctly wider than postsoma	***Aulacaspis ericacearum* Takagi**
33	L1 sunken into the apex of pygidium, forming a large notch at the apex of the pygidium; anterior spiracles each with 16 trilocular pores; only one submedial macroducts on abdominal segment VI	***Aulacaspis yasumatsui* Takagi**
–	L1 almost parallel on inner basal margins, then strongly divergent to their apices; anterior spiracles each with 30–50 trilocular pores; with 2-4 submedial macroducts on abdominal segment VI	***Aulacaspis machili* (Takahashi)**
34	Submedial dorsal macroducts present on abdominal segment I, forming a double row	**35**
–	Submedial dorsal macroducts present or absent on abdominal segment I; if present, forming single row	**41**
35	Submedial dorsal macroducts present on segment VI, forming double or triple row	***Aulacaspis murrayae* (Takahashi)**
–	Submedial dorsal macroducts present or absent on segment VI; if present, forming a single row	**36**
36	Submarginal dorsal macroducts present on abdominal segment II, forming a double row	**37**
–	Submarginal dorsal macroducts present on abdominal segment II, forming a single row	**38**
37	Both submedial and submarginal dorsal macroducts present on abdominal segment I	***Aulacaspis actinidiae* Takagi**
–	Both submedial and submarginal dorsal macroducts absent from abdominal segment I	***Aulacaspis spinosa* (Maskell)**
38	Submarginal dorsal macroducts present on abdominal segment I	***Aulacaspis citri* Chen**
–	Submarginal dorsal macroducts absent from abdominal segment I	**39**
39	Submarginal dorsal macroducts present on abdominal segment II and III, forming double row, gland tubercles present on segment I	***Aulacaspis intermedius* (Chen, Wu & Su)**
–	Submarginal dorsal macroducts present on abdominal segment II and III, forming single row, gland tubercles absent from segment I	**40**
40	L1 projecting beyond apex of pygidium; anterior spiracles each with 40–60 trilocular pores, posterior spiracles each with 20–30 trilocular pores	***Aulacaspis projecta* Takagi**
–	L1 sunken into the apex of pygidium; anterior spiracles each with less than 30 trilocular pores, posterior spiracles each with 2–7 trilocular pores	***Aulacaspis crawii* (Cockerell)**
41	Dorsal macroducts forming a double row on submedial area of abdominal segment I	**42**
–	Dorsal macroducts forming a single row on submedial area of abdominal segment I	**46**
42	Dorsal macroducts forming a double row on submedial area of abdominal segment IV	**43**
–	Dorsal macroducts forming a single row on submedial area of abdominal segment IV	**44**
43	Prosomatic tubercles robust; L1 parallel on inner basal margins, then strongly divergent to their apices	***Aulacaspis rosarum* (Borchsenius)**
–	Prosomatic tubercles not discernible; L1 sunken into the apex of pygidium, forming a large notch at the apex of the pygidium	***Aulacaspis megaloba* Scott**
44	L1 sunken into the apex of pygidium, forming a large notch at the apex of the pygidium	***Aulacaspis litseae* Tang**
–	L1 almost parallel on inner basal margins, then strongly divergent to their apices	**45**
45	Prosomatic tubercles robust; with 4–5 dorsal macroducts on submarginal area of abdominal segment V	***Aulacaspis guangdongensis* Chen, Wu & Su**
–	Prosomatic tubercles not discernible; with 1 dorsal macroduct on submarginal area of abdominal segment V	***Aulacaspis longanae* Chen, Wu & Su**
46	Dorsal macroducts forming a double row on submedial area of abdominal segment III	**47**
–	Dorsal macroducts forming a single row on submedial area of abdominal segment III	**50**
47	Dorsal macroducts present on abdominal segment I	**48**
–	Dorsal macroducts absent from abdominal segment I	**49**
48	Prosomatic tubercles robust; marginal macroducts between L1 and L2 longer than the length of L1; inner margin of L1 slightly serrate	***Aulacaspis greeni* Takahashi**
–	Prosomatic tubercles not discernible; marginal macroducts between L1 and L2 equal or shorter than the length of L1; inner margin of L1 not serrate	***Aulacaspis phoebicola* Takahashi**
49	Dorsal macroducts present on submedial area of abdominal segment II, forming double row; anterior spiracles with 30 trilocular pores	***Aulacaspis acronychiae* Takagi & Martin**
–	Dorsal macroducts present on submedial area of abdominal segment II and IV, forming single row; anterior spiracles with 15 trilocular pores	***Aulacaspis thoracica* (Robinson)**
50	Dorsal macroducts present on submedial area of abdominal segment VI	51
–	Dorsal macroducts absent from submedial area of abdominal segment VI	***Aulacaspis neospinosa* Tang**
51	Both submedial and submarginal dorsal macroducts present on abdominal segment I	***Aulacaspis divergens* (Takahashi)**
–	Both submedial and submarginal dorsal macroducts absent from abdominal segment I	***Aulacaspis maesae* Takagi**

## Supplementary Material

XML Treatment for Aulacaspis

XML Treatment for Aulacaspis zunyiensis
